# Supplementary figures and images for: Human osteoclastogenesis in Epstein-Barr virus-induced erosive arthritis in humanized NOD/Shi-scid/IL-2Rγnull mice
Source: PLoS One. 2021 Apr 1;16(4):e0249340. doi: 10.1371/journal.pone.0249340 (PMC8029598; doi:10.1371/journal.pone.0249340)

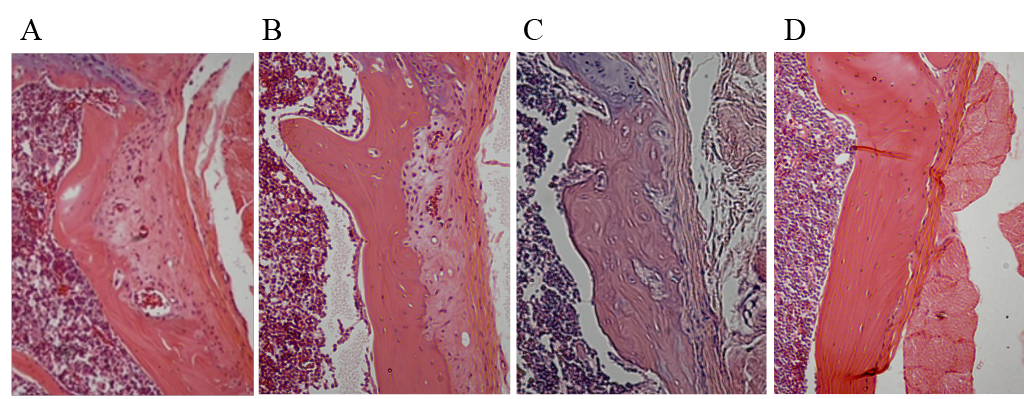

Supplement: S1 Fig — Knee joint sections. (A) Severe bone erosion, defined as grade 3+, in an EBV-infected mouse. (B) Mild bone erosion, defined as grade 2+, in an EBV-infected mouse. (C) Slight bone erosion, defined as grade 1+, in an EBV-infected mouse. (D) Lack of bone erosion in an uninfected mouse. Original magnification, 100×. (TIF) [file pone.0249340.s001.tif]

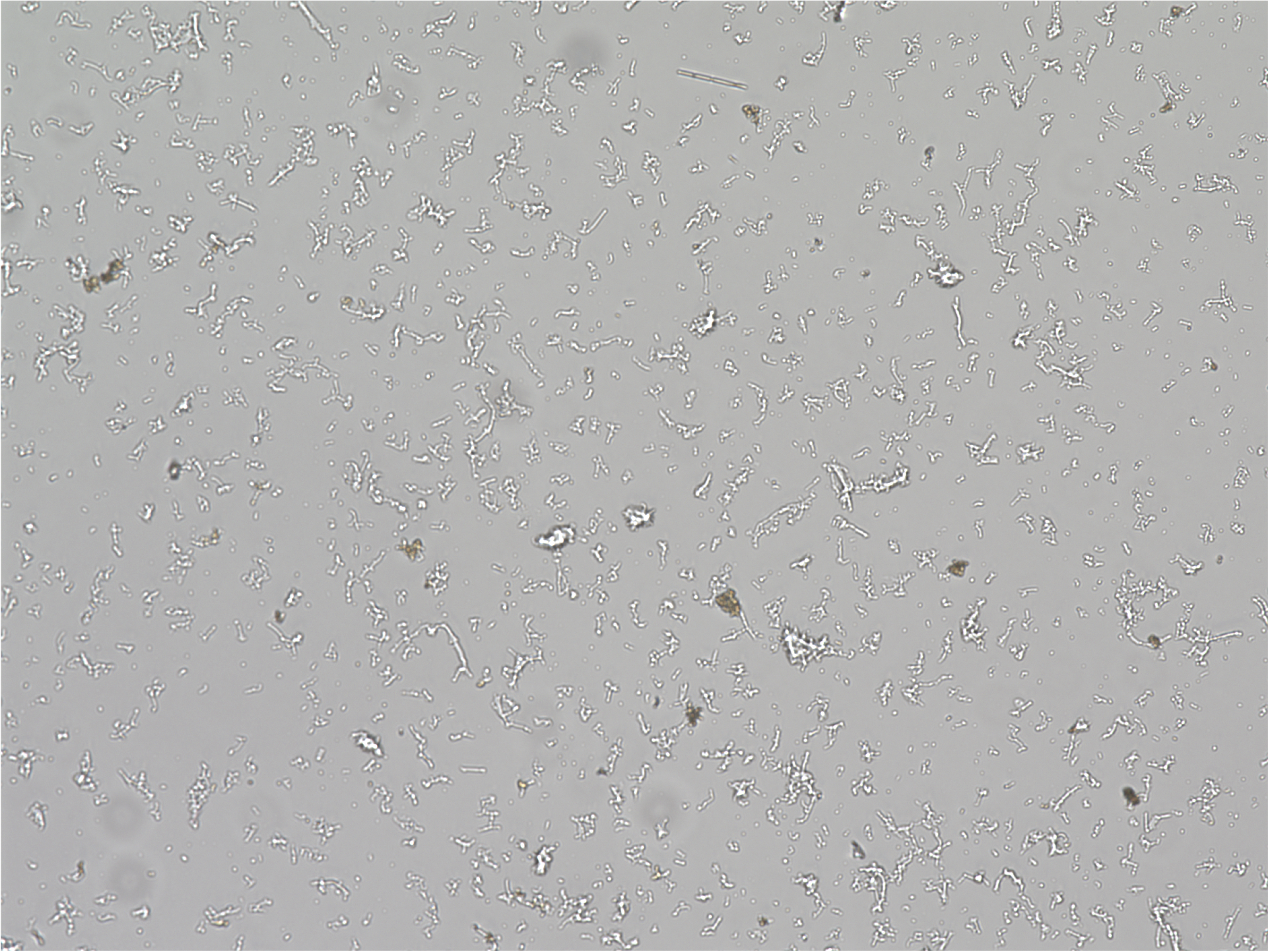

Supplement: S2 Fig — Adherent cells with a tendency to develop into osteoclasts in vitro could not be cultured. Original magnification, 40×. (TIFF) [file pone.0249340.s002.tiff]

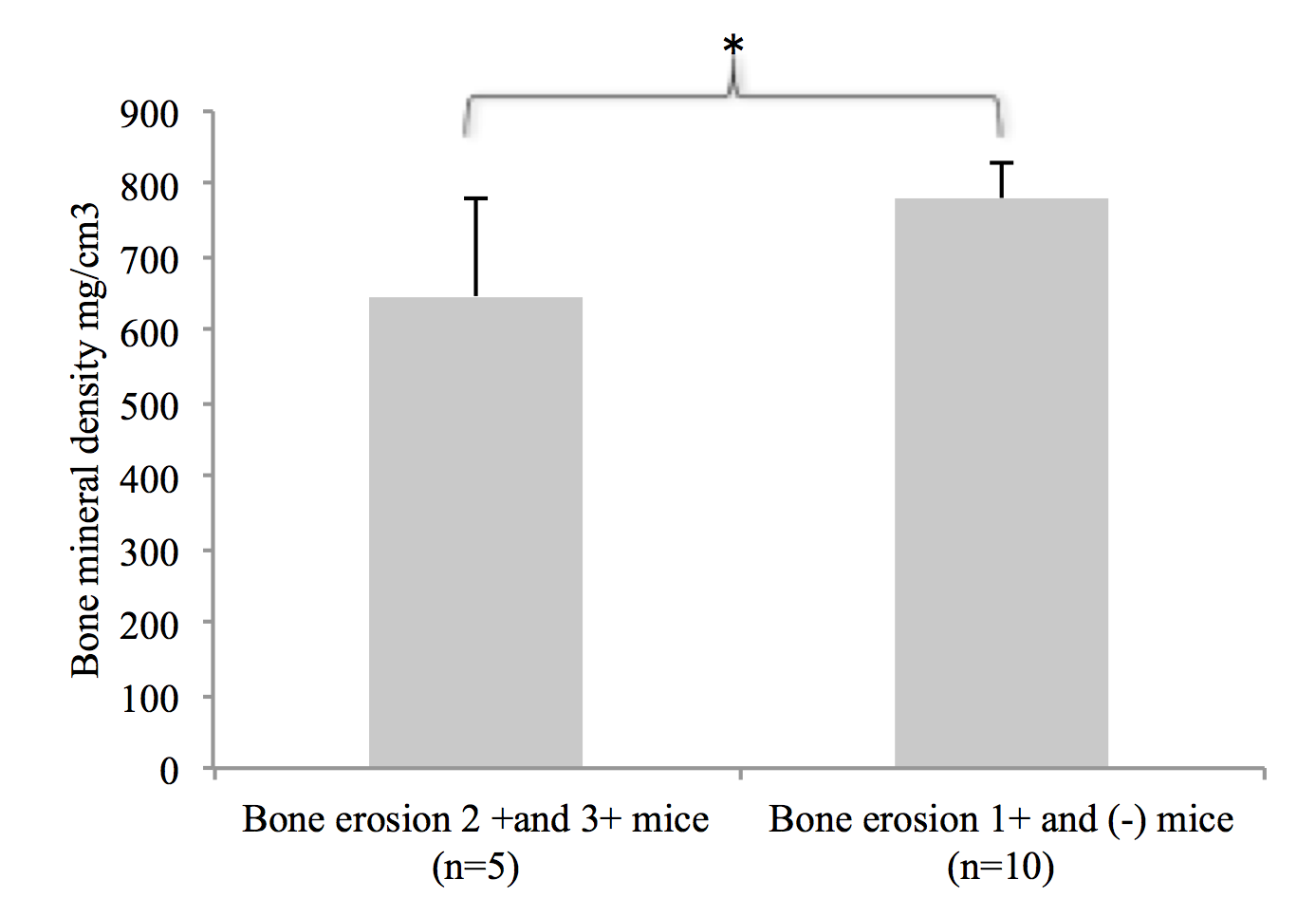

Supplement: S3 Fig — Bone mineral density was compared between EBV-infected mice having 3+ or 2+ bone erosion (n = 5) and EBV-infected mice having 1+ bone erosion (n = 5) or EBV-uninfected mice having no bone erosion (n = 5). Severe bone erosion group had significantly lower BMD grades than those of mild erosion group. Values represent mean ± standard deviation. Statistical analysis was performed using student’s t-test (*P value = 0.0439). (TIFF) [file pone.0249340.s003.tiff]
