# Supplementary material for: Human osteoclastogenesis in Epstein-Barr virus-induced erosive arthritis in humanized NOD/Shi-scid/IL-2Rγnull mice
Source: PLoS One. 2021 Apr 1;16(4):e0249340. doi: 10.1371/journal.pone.0249340 (PMC8029598; doi:10.1371/journal.pone.0249340)
Supplement: Attachment — Submitted filename: Response_to_Reviewers.docx [file pone.0249340.s004.docx]

*Journal Requirements:*

*When submitting your revision, we need you to address these additional requirements.*

*1. Please ensure that your manuscript meets PLOS ONE's style requirements, including those for file naming.*

 Response: Thank you for your helpful suggestions. We have ensured that PLOS ONE’s style requirements have been met.

*2.In your submission, you state that your work was presented in abstract form at the 2014 american college of rheumatology annual meeting.
Please clarify whether this abstract is under copyright, and whether the copyright owner is the authors or the college of rheumatology. If the copyright is not held by the authors, permission from the original copyright holder to publish under a CC BY license needs to be obtained and the following should be completed:*

Response: This abstract is not under copyright. The American College of Rheumatology (ACR) states the copyright policy as follows: ‘The information and materials displayed and presented “during this meeting” are the property of the ACR and the presenter and cannot be photographed, copied, photocopied, transformed to electronic format, reproduced, or distributed without written permission of the ACR and the presenter.’

https://acrabstracts.org/download-abstracts/

*3. Please provide the dilutions of all antibodies used in your study.*

Response: All antibody dilution ratios have been added to the manuscript.

*4. Please include further information regarding your animal research, per our guidelines (*[*http://journals.plos.org/plosone/s/submission-guidelines#loc-animal-research*](http://journals.plos.org/plosone/s/submission-guidelines#loc-animal-research)*). Specifically, please provide details regarding:
 1) Animal health monitoring, including:
     -frequency of monitoring,
     -monitoring criteria, and
     -any efforts made to reduce suffering and distress, such as administering
      analgesics.
 2) any mortality that occurred outside of planned euthanasia
 3) The total number of mice.*

Response: We added further information about the above 1) to 3) in the manuscript.

*5. We note that you have included the phrase “data not shown” in your manuscript. Unfortunately, this does not meet our data sharing requirements. PLOS does not permit references to inaccessible data. We require that authors provide all relevant data within the paper, Supporting Information files, or in an acceptable, public repository. Please add a citation to support this phrase or upload the data that corresponds with these findings to a stable repository (such as Figshare or Dryad) and provide and URLs, DOIs, or accession numbers that may be used to access these data. Or, if the data are not a core part of the research being presented in your study, we ask that you remove the phrase that refers to these data.*

Response: We added supporting information about bone mineral density as S3 Fig. Additionally, we removed the description about the number of human osteoclast progenitor cells because we decided that more detailed data were needed for the discussion.

*Reviewer #1: Human osteoclastogenesis in Epstein-Barr virus-induced erosive arthritis in humanized NOD/Shi-scid/IL-2Ryneull mice by Nagasawa et al describes a humanized mouse model in which erosive arthritis during EBV infections can be studied. Their results suggest that human osteoclasts induce erosive arthritis during EBV infection.*

*This is exciting and significant work but proper controls need to be shown. Without the necessary controls this review can’t properly evaluate the quality of the data. These controls should be added in addition to increasing the number of representative pictures and sample sizes as described below.*

Response: Thank you for your constructive criticism and helpful suggestions. We have evaluated our antibodies on murine osteoclasts and confirmed that they do not react with mouse osteoclasts. This indicates that those osteoclasts detected in the arthritis lesions of EBV-infected humanized mice are truly of human origin. In addition, we have shown data from additional mice that support our conclusion. We believe our revised manuscript provides more concrete evidence of human osteoclast differentiation in EBV-infected humanized mice and their involvement in RA-like erosive arthritis.

Point by point response

*Results section. Page 18. Outline the and describe the experiment broadly before jumping into the specific results to make it easier for the reader to follow.*

Response: Our previous work demonstrated consistent and rapid increase in the number of peripheral blood CD8^+^ T cells and reversal of CD4/CD8 ratio in EBV-infected humanized NOG mice, which constitute the flow-cytometric hallmarks of successful EBV infection. This study starts with reproduction of these results and then goes on to further characterize bone erosion induced by EBV. To make this outline clearer, we have added the following sentence at the beginning of the Results.

“In this study, we initially confirmed our previous findings of rapid increase in the number of CD8^+^ cells and the reversal of CD4/CD8 ratio in the peripheral blood of humanized mice following EBV infection.”

*Figure 1. The legend needs to be more descriptive. Are these averages of the 10 infected samples?*

*Error bars need to be included and statistics should be provided. The data for the uninfected samples needs to be included.*

Response: We have revised Figure 1 legend and described the experiment in more detail. The data shown in this figure are not the average of 10 mice but those from a representative mouse. In a previous publication, we demonstrated rapid increase in the number of peripheral blood CD8^+^ T cells and the reversal of CD4/CD8 ratio as very consistent responses following EBV infection of humanized mice (Yajima et al, J Infect Dis 198:673–682, 2008). Figure 1 depicts the outline of changes in the profile of human lymphocytes following EBV infection, and the indicators to confirm that our humanized NOG mice are certainly infected with EBV. The data from uninfected mice were shown in a previous publication (Yajima et al, J Infect Dis 198:673–682, 2008), which indicated that while the percentage of CD3+ T cells among peripheral human CD45+ leukocytes increased gradually, that of CD19+ B cells decreased. The CD4/CD8 ratio remained >1 always.

The revised Figure 1 legend: “Fig 1. Time course of human lymphocyte reconstitution in the peripheral blood of an EBV-infected hu-NOG mouse. The percentage of human CD45^+^, CD4^+^, CD8^+^, or CD19^+^ cells among the peripheral blood mononuclear cells was measured weekly, after inoculation with EBV. Upon EBV infection, the percentage of human CD8^+^ T cells increased rapidly and the ratio of CD4^+^ cells to CD8^+^ cells decreased to below 1. Data from a representative mouse are shown.”

*Table 1. Why were different numbers of CD34+ cells used for different sets? What is the rational for different amounts?*

Response: In our previous publication, we showed that transplantation of 1 × 10^4^ to 1.2 × 10^5^ cells/mouse of CD34+ HSCs resulted in consistent reconstitution of human lymphocytes and monocyte/macrophages. In this study, the number of HSCs/mouse was varied within this range, depending on the number of available mice and available HSCs. The efficiency of immune reconstitution was not affected by this variation of HSCs input.

*Figure 2. Images are only shown for 1 infected and 1 uninfected mouse. More representative pictures should be added. I would like to see pictures representing the different severity of bone erosion described in Table 1. (ie samples from 1, 2, and 3+ in addition to the negative control).*

Response: We have added pictures of another infected mouse and from other angles in the revised manuscript. We could afford to use only 2 infected mice and 2 uninfected mice in three-dimensional computed tomography, because other mice were examined through other analyses. This figure, however, clearly shows bone destruction lesions similar to those revealed in patients with RA using the same diagnostic method. Typical examples of bone erosion 1+, 2+, and 3+ in addition to the negative control in the knee joint tissue are shown in Supplementary Figure S1.

*Figure 3. Only 2 mice from EBV infected or uninfected where examined. 10 mice were infected – including more samples would increase the significance. Only one picture is shown for TRAP, Cathepsin and MMP-9 staining. The authors state these antibodies do not react with the mouse proteins but the negative controls are necessary and need to be shown.*

Response: In the revised Figure 3, we show immunohistochemical data from an additional mouse that shows the same results. We have obtained mouse osteoclasts by culturing mouse osteoclast progenitor cells with mouse M-CSF and mouse RANKL. These mouse osteoclasts did not react with the antibodies against human MMP-9 and mitochondria, while they were stained positively by antibodies specific for murine MMP-9 and mitochondria, indicating that those multinuclear cells found in EBV-infected humanized mice are truly of human origin. The results are shown in the revised Figure 5. Regarding staining with anti-cathepsin K antibody, additional experiments could not be planned because the antibody had been discontinued.

*Figure 4. An n=2 is low and only single pictures are shown. Negative controls are necessary and should be shown.*

Response: We have shown results from 3 additional mice in the revised Figure 4. We could obtain consistent results from these two mice, and therefore, we think the results are reliable. As stated in the reply to the previous comment, we have shown that our antibodies did not react with murine osteoclasts and the results are shown in Figure 5.

*Figure 5. n=3 but only one sample is shown.*

Response: In the revised Figure 6, we present data from 2 additional mice that show the same result.

*Supplemental figure doesn’t have a legend.*

Response: We have revised the Figure S2 legend and described the experiment in more detail.

*Reviewer #2: The article “Human osteoclastogenesis in Epstein-Barr virus-induced erosive arthritis in humanized NOD/Shi-scid/IL-2Rgnull mice” is an interesting original study that starts to elucidate the mechanisms of EBV-induced erosive arthritis through a hu-NOG model. The study aims are addressed by its methodology and the three-dimensional computed tomography was a visually appealing tool to demonstrate bone erosive changes. The presentation of results is linear and the data is properly discussed, supporting the authors conclusions. Although this is true, there are some unanswered questions and revisions that will make this study more impactful.*

Response: We thank you for your favorable comments and helpful suggestions. We have included the data for reconstruction of human CD19^+^ B cells in Figure 1. We have also modified the Materials and methods to correct our inconsistent statements concerning histochemistry. We believe that the manuscript has been sufficiently improved, and the revised manuscript supports our conclusion adequately.

Point by point response

*Major Comments*

*The authors do not show in vitro osteoclast differentiation of bone marrow cells (cultured with human RANKL and M-CSF) from EBV-uninfected NOG (hu-NOG only).*

*Even though they say that EBV infection did not increase the number of osteoclast progenitors (in a data not shown - sentence line 440-446) they do not provide evidence that EBV-uninfected hu-NOG have human osteoclast progenitor cells in their bone marrow, or that these progenitors are able to differentiate in osteoclast in vitro as for the EBV-infected hu-NOG.*

*I believe that if the authors provide the data showing in vitro osteoclast differentiation of bone marrow cells from EBV-uninfected hu-NOG, cultured in the presence of human RANKL and human M-CSF it would demonstrate potential for differentiation as well as equal progenitor*

Response: In response to reviewer’s comment, we cultured bone marrow cells of EBV-uninfected hu-NOG mice in the presence of human M-CSF and human RANKL differentiation signals. As the reviewer pointed out, EBV-uninfected hu-NOG mice have human osteoclast progenitor cells in their bone marrow.

*Typically in normal bone remodeling there is a balance between osteoblasts and osteoclasts, with the former being responsible for inducing the differentiation of the latter through the production of RANK-L and M-CSF. It’s true that in pathogenic settings the osteoblasts are not the main sources of these cytokines, but they are still important for conferring some osteoprotection. Although the authors bring this to their discussion, there is no information on osteoblasts in their data. It would be informative to understand the ratio of osteoblast to osteoclasts in hu-NOG and after infection with EBV.*

Response: We agree with the reviewer that the osteoblast/osteoclast ratio is important for bone homeostasis, but we have not yet examined the ratio in our EBV-infected mice. However, we believe our data are sufficient to claim that EBV infection of the humanized mice resulted in the differentiation of human osteoclasts that are most likely responsible for bone erosion found in these mice.

*Minor comments*

*In the methods section the authors list CD19+ as one of the monitored percentages of human cells in the blood of hu-NOG. In Fig. 1 the authors do not show CD19+ numbers, although one can assume by subtracting CD4+ and CD8+ from total Human CD45+, it would be easier to observe how the frequency of these cells stay through the experiment course if a CD19+ curve was displayed.*

Response: We have shown the CD19^+^ curve in the revised Figure 1.

*Fig. 5 - it appears that the cells stained for TRAP (in Fig. 5A and B) were also stained for hematoxylin, correct? If that is true, since this was performed in a glass bottom dish, the statement in line 219 of the methods section “After washing, only the plates of serial sections were stained in hematoxylin (cultured cells on the chamber slides, pit formation assay plates, and glass bottom dishes were not stained” is incorrect.*

Response: We have revised the Methods section and restated that “After washing, serial sections in the plates and cultured bone marrow cells of pelvic bones in glass bottom dishes were stained with hematoxylin (cultured cells on the chamber slides and pit formation assay plates were not stained).”

*Line 645 - Possible typo/missing verb “be cultured”*

Response: We have revised as suggested.

*Have the authors considered staining for collagen (with picrosirius red staining or by using polarized light microscopy) to assess cathepsin K effects in the joints of EBV-infected hu-NOG.*

Response: We have not stained for collagen in our mouse samples, although we expect to see degradation. We plan to perform further immunohistochemical characterization of our mouse arthritis model during the next stage of our research.

*Aside RANKL and M-CSF there are a number of inflammatory cytokines that are usually present in inflamed joints that impact bone remodeling within that microenvironment. Given that the bone marrow cells of EBV-infected hu-NOG when cultured in vitro (without human RANKL and M-CSF cytokines) resulted in osteoclast differentiation and the authors had previously shown bone marrow edema in EBV-infected mice, one could assume that pro-inflammatory cytokines have an important role in this process. Specially since T cells do not survive for long in culture, even with proper stimuli, cells from the monocytic/macrophage lineage could be the sources of this cytokines in this case. Have the authors investigated IL-1 (alpha and or betta) and TNF-a (that have been previously implicated in osteoclastogenesis), in the bone marrow of EBV-infected hu-NOG?*

Response: We have recently initiated the analysis on the mechanism of osteoclast differentiation induced by EBV and believe it is necessary to examine the role of human inflammatory cytokines, including IL-1 and TNF-α. We have not performed the experiments yet.
