# Supplementary material for: Human osteoclastogenesis in Epstein-Barr virus-induced erosive arthritis in humanized NOD/Shi-scid/IL-2Rγnull mice
Source: PLoS One. 2021 Apr 1;16(4):e0249340. doi: 10.1371/journal.pone.0249340 (PMC8029598; doi:10.1371/journal.pone.0249340)
Supplement: Attachment — Submitted filename: Rsponse to Reviewers.docx [file pone.0249340.s005.docx]

*Reviewer #1: The authors have addressed my initial concern but I still have a few comments and suggestions.

The authors added the statement “In this study, we initially confirmed our previous findings of rapid increase in the number of CD8+ cells and the reversal of CD4/CD8 ratio in the peripheral blood of humanized mice following EBV infection.” to the beginning of the results section. Please provide a reference.*

Response: Thank you for your helpful suggestions. We added a reference number at the beginning of the results section properly.

*The authors state the information in figure 1 is not an average bu a representation from 1 mouse. I think showing the average with error bars would give a representation of how consistent the results were.*

Response: Based on your feedback, we revised figure 1 and showed the average values of EBV-infected mice with error bars in the figure.

*Figure S1 is labeled in this order A,C, B, D. Is this intentional or is A, B, C, D meant.*

Response: As you pointed out, these labels ware inappropriate. We changed these labels appropriately. A, C, B, D are incorrect, A, B, C, D are correct.

*Figures 3 ,4 and 6 would be easier to read if labels such as A1, A2, B1, etc were replaced with the antibody that was used for staining.*

Response: As you pointed out, these labels were difficult to understand. A1, A2, B1, etc in these labels ware replaced with the name of the stained antigen.

*Figure 3 A1, A2. Are these the same picture at different magnifications? When multiple pictures are shown for the the same antibody stain please indicate if it is from the same mouse. The figures would be enhanced with a more descriptive labeling scheme.*

Response: Yes, images of A1 and A2 in the figure 3 are same picture at different magnifications. Based on your feedback, we revised the figure and indicated mouse numbers in the labels.

*Reviewer #2: The introduction of additional images to Figures 2; 3; 4 and 6, were a good improvement of the manuscript, strengthening the authors findings.
However, figure labeling could be improved to ensure better readability. The current panel labeling (“A-1; B-1”) and the amount of panels in each figure, are hard for readers to follow without constantly looking at the figure legend.
The authors could identify the panels with the molecule they are showing on top in the vertical orientation while identifying the panels with the correct experimental group in the horizontal. That would also potentially improve the figure legend itself.*

Response: Thank you for your favorable comments and helpful suggestions. As you pointed out, these figures were difficult to understand. Based on your feedback, we showed the names of the stained antigens and mouse number at the top and side of their panels in these figures.
